# Extreme Outliers in Lower Stratospheric Water Vapor Over North America Observed by MLS: Relation to Overshooting Convection Diagnosed From Colocated Aqua‐MODIS Data

**DOI:** 10.1029/2020GL090131

**Published:** 2020-12-19

**Authors:** F. Werner, M. J. Schwartz, N. J. Livesey, W. G. Read, M. L. Santee

**Affiliations:** ^1^ Jet Propulsion Laboratory California Institute of Technology Pasadena CA USA

**Keywords:** water vapor, MLS, MODIS, stratosphere, retrieval, overshooting convection

## Abstract

Convectively injected water vapor (H_2_O) in the North American (NA) summer lowermost stratosphere results in significant outliers in the 100‐hPa H_2_O measurements from the Aura Microwave Limb Sounder (MLS). MLS statistics from 15 years confirm that the NA region contains over 60% of global 100‐hPa H_2_O > 12 ppmv, despite having only ∼1.8% of all MLS observations. A profile sampled in August 2019 stands out, with $H_{2}O=26.3$ ppmv, far exceeding the prior record and the median ∼4.5‐ppmv abundance in NA. This particular outlier is associated with a large overshooting convective event (OCE) that spanned multiple U.S. states and persisted for several hours. Colocation of the MLS data over NA with cloud observations from Aqua's Moderate Resolution Imaging Spectroradiometer (MODIS) reveals the unique character of this case, as only 2.3% of MLS profiles are as close to an OCE and only 0.024% of OCEs cover as large an area within a 500‐km perimeter of a profile.

## Key Points

- A profile with unprecedented stratospheric humidity is associated with large overshooting convection
- Large lower stratospheric humidity outliers are in close proximity to strong overshooting convection
- MLS humidity indicates trend toward larger stratospheric outliers and strengthening of North American monsoon

## 1 Introduction

Stratospheric water vapor (H_2_O) has long been known to impact the atmospheric radiation budget (Dvortsov & Solomon, 2001), as well as stratospheric ozone (Solomon et al., 1986). Studies (e.g., Dessler et al., 2013; Solomon et al., 2010; Wang et al., 2017) illustrate that H_2_O increases in the upper troposphere and lower stratosphere (UTLS) can lead to substantial surface warming. A detailed understanding of the processes controlling UTLS H_2_O is essential for understanding water vapor's roles in climate feedbacks and for improving climate projections.

Previous studies indicate that overshooting convective events (“OCEs”) can substantially impact stratospheric H_2_O concentrations (e.g., Avery et al., 2017; Dessler & Sherwood, 2004; Grosvenor et al., 2007; Randel et al., 2015; Schoeberl et al., 2014; Ueyama et al., 2018; Wang et al., 2019). In such events, ice particles are lofted to altitudes above the cold‐point tropopause, where they can sublimate and moisten the lowermost stratosphere (LMS) (e.g., Corti et al., 2008; Nielsen et al., 2007). Such convective moistening contributes to regional H_2_O maxima in the LMS over the boreal summer monsoon regions (e.g., Anderson et al., 2012; Carminati et al., 2014; Dessler & Sherwood, 2004; Randel et al., 2001; Smith et al., 2017). However, quantifying the relative importance of OCEs and the poleward advection of high humidity (Rosenlof et al., 1997) is the subject of ongoing studies. Since OCEs contribute to stratospheric humidity globally, projections of increased convection in a warmer climate imply a potential positive climate feedback via stratospheric humidity.

Using global Version 3 observations from the Aura Microwave Limb Sounder (MLS) over the years 2005–2012, Schwartz et al. (2013) (“S2013” hereinafter) revealed that OCEs over the North American monsoon area (“NA”) yield elevated H_2_O in the LMS in up to 1% of profiles. While numerous studies discuss localized negative correlations between OCEs and ozone concentrations (e.g., Anderson et al., 2012; Héron et al., 2020; Solomon et al., 2005), the overall impact of OCEs on stratospheric ozone is currently believed to be low (e.g., Homeyer et al., 2014; Robrecht et al., 2019; Schwartz et al., 2013; World Meteorological Organisation, 2018).

Our study reports updated statistics on MLS 100‐hPa H_2_O, adding 7 years (2013–2019) of data and using Version 4 products. This 15‐year record not only yields more reliable H_2_O retrievals to identify global regions of H_2_O outliers in the LMS, but also indicates an increase in the frequency of those outliers over NA. A case study of one OCE with the highest 100‐ to 83‐hPa H_2_O in the MLS record is presented, as well as an examination of a 15‐year record of colocated MLS 100‐hPa H_2_O and cloud properties from the Moderate Resolution Imaging Spectroradiometer (MODIS), which permits the inference of correlations between OCE metrics and large outliers in stratospheric humidity. Taken together, our analyses illustrate the connection between OCEs and H_2_O outliers in the LMS over NA, which appear to occur more frequently and earlier in the year in 2013–2019 than in 2005–2012.

## 2 Data

MLS (Waters et al., 2006) measures ∼3,500 profiles per day. Here we use Version 4.2x H_2_O (Livesey et al., 2020). This updated data set provides more reliable H_2_O retrievals, especially in the UTLS and in the presence of high clouds. MLS lower stratospheric H_2_O has a vertical resolution of 3 km and a resolution of ∼200 km by 3 km along and across the orbital track, respectively. This study focuses on 100 hPa, where the precision of individual H_2_O measurements is ∼0.4 ppmv.

To evaluate convection upstream of each MLS profile, we use Lagrangian Trajectory Diagnostics (Livesey et al., 2015) that have been updated to use winds and diabatic heating rates from the Modern‐Era Retrospective Analysis for Research and Applications, Version 2 (MERRA‐2) data set (Gelaro et al., 2017).

MLS H_2_O profiles are colocated with cloud top properties retrieved from measurements made by two different instruments: (i) The Advanced Baseline Imager (ABI) aboard GOES‐R and (ii) Aqua‐MODIS. ABI samples data in 16 channels from the visible to the infrared, providing a full disk hemispheric image every 10 minutes (Greenwald et al., 2016). These radiances yield retrievals of cloud top pressure (*p*_CT_), cloud optical thickness (*τ*), effective particle radius (*r*_eff_), and cloud phase (Minnis, 2007), all available at horizontal scales of 2–10 km at nadir. Continuous GOES‐R cloud products are only available since 2019. However, a longer‐term data set of cloud variables is provided by MODIS, which precedes Aura in orbit by ∼15 min. MODIS views a 2,330‐km‐wide swath, providing global coverage every 2 days. The spatial resolution at nadir is 1 km for a majority of the 36 spectral bands and reported cloud products; the pixel size increases toward the swath edges. We use MODIS‐derived *p*_CT_, *τ*, *r*_eff_, and cloud phase from Data Collection 6.1 (Ardanuy et al., 1992; Barnes et al., 1998; Platnick et al., 2003).

In order to detect OCEs, values of *p*_CT_ are related to the temperature (*T*_TP_) and pressure (*p*_TP_) at the tropopause provided by MERRA‐2 at 1 hr and 0.5° × 0.625° resolution. The tropopause is determined using the “blended estimate” from Wilcox et al. (2012). The cloud products from GOES‐R and MODIS are subsequently used to calculate secondary cloud characteristics. The tropopause‐relative cloud top altitude (*δz*) is estimated from the pressure difference $\delta p=p_{\text{CT}}-p_{\text{TP}}$ by assuming hydrostatic equilibrium:

(1) $$\delta z=-1\cdot \frac{\delta p}{\rho_{\text{air}}\cdot g},\;\;\text{with}$$

(2) $$g=9.80665\cdot {\left(\frac{R_{e}}{R_{e}+z}\right)}^{2},\;\text{and}$$

(3) $$\rho_{\text{air}}=\frac{p_{\text{CT}}}{R\cdot T_{\text{TP}}}.$$

Here, *g* is the acceleration of gravity (assumed constant here), *R*_e_ = 6,367,000.445 m is the Earth's average radius, *z* = 15,000 m is the average altitude assumed in this study, *ρ*_air_ is the air density at the tropopause, and R=287.058 J kg^−1^ K^−1^. Another secondary cloud variable is the ice water path (*W*_i_), which is proportional to the product of retrieved *τ* and *r*_eff_ (e.g., Brenguier et al., 2000; Miller et al., 2016).

## 3 Update on High H_2_O in the Global MLS Record

**Figure 1:**
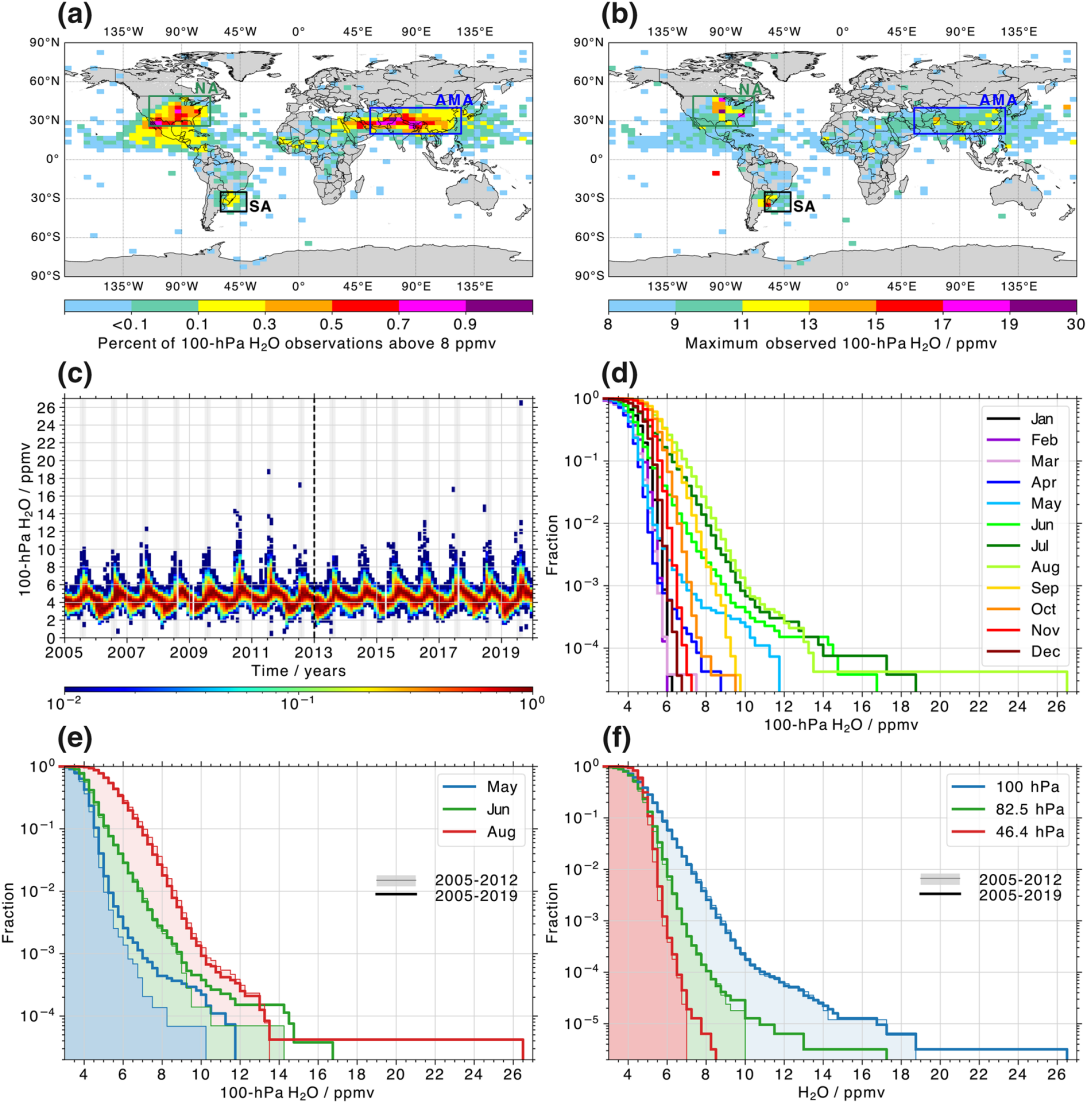
(a) Frequency with which MLS 2005–2019 100‐hPa H_2_O mixing ratios exceed 8 ppmv. Light blue colors represent a 3° × 5° bin containing a single observation. Contributions from two exceptional pyrocumulonimbus injection events in 2009 (Pumphrey et al., 2011) and 2017 (Torres et al., 2020), as well as the Kasatochi (August 2008) and Calbuco (April 2015) volcanic eruptions (Sioris et al., 2016), have been excluded from this analysis (by excising all observations in the associated calendar month across the affected hemisphere). (b) Similar to (a), but showing the maximum 100‐hPa H_2_O. Note the jump in contour levels between 8–9 and 9–11 ppmv, as well as 17–19 and 19–30 ppmv. (c) Time series of monthly histograms (each normalized by its maximum) of 100‐hPa H_2_O in the NA box over 2005–2019, again excluding pyrocumulonimbus and volcanic impacts. Gray shading indicates July and August, while the vertical, dashed line highlights the transition from 2005–2012 to 2013–2019. (d) Monthly probabilities that 100‐hPa H_2_O observations exceed a given mixing ratio. (e) Same as (d), but comparing May‐August probabilities between the earlier years (2005–2012; translucent shading) and the full study period (2005–2019; solid lines). (f) Probabilities that 100‐ to 46.4‐hPa H_2_O exceed a given mixing ratio over 2005–2012 (translucent shading) and 2005–2019 (solid lines). Panels (a)–(d) were adapted and updated from Figures 2 and 3 in S2013.

Figure 1a illustrates the global distribution of 100‐hPa H_2_O > 8 ppmv over 2005–2019. This threshold from S2013 corresponds to the 99.9th percentile of H_2_O at that level. Figure 1b shows the maximum observed 100‐hPa H_2_O within each 3° × 5° (latitude × longitude) box over the same period. Three areas, defined in S2013, are notable for H_2_O outliers in the LMS: the NA region (green box; 68–115°W, 26–49°N), a smaller area in South America (black box labeled “SA”; 40–60°W, 25–40°S), and the Asian summer monsoon area (blue box labeled “AMA”; 55–125°E, 20–40°N). These regions contain 1.8%, 0.5%, and 2.2% of the total MLS profiles, respectively. With the updated Version 4 products, the NA region appears to extend further over Central America than seen in S2013. OCE influence in this area has been reported (e.g., Clapp et al., 2019); however, H_2_O outliers at 100 hPa just barely exceed 8 ppmv (light blue color in Figure 1b), and the region is almost devoid of outliers with an adjusted threshold of H_2_O > 8.5 ppmv.

Percentiles of H_2_O at 100 and 82.5 hPa within the three regions, as well as the percentages of global MLS profiles larger than certain H_2_O thresholds, are provided in Table 1. While both NA and AMA contain a sizable fraction of H_2_O > 8 ppmv, successively larger mixing ratios are increasingly concentrated over NA relative to all other regions, including a remarkable 100‐hPa maximum value of 26.3 ppmv (the next largest 100‐hPa maximum in the NA box is 18.6 ppmv). That same profile, sampled on 27 August 2019 and indicated by the single purple grid box in Figure 1b, is also a new NA maximum of 17.0 ppmv at 82.5 hPa (the next largest being 12.9 ppmv) and exceeds the 99th percentile for H_2_O at 147, 121, and 68 hPa in the NA region (not shown).

**Table 1:** Percentiles and Maximum Values of MLS 100‐ and 82.5‐hPa H_2_O Within the NA, AMA, and SA Boxes, Shown in Figures 1a and 1b, and Those From All Other Areas; Moreover, the Percentages of Global H_2_O > 8, 10, 12, 14, 16, and 18 ppmv Within Each Region Are Given

	100 hPa				82.5 hPa			
	NA	AMA	SA	Other	NA	AMA	SA	Other
1st perc. (ppmv)	2.7	2.2	2.6	1.9	2.6	2.2	2.7	1.7
10th perc. (ppmv)	3.4	3.0	3.2	3.0	3.4	3.0	3.2	2.8
25th perc. (ppmv)	3.9	3.6	3.6	3.7	3.9	3.5	3.6	3.6
50th perc. (ppmv)	4.5	4.3	4.1	4.2	4.3	4.2	3.9	4.1
75th perc. (ppmv)	5.1	5.1	4.6	4.6	4.7	4.8	4.3	4.5
90th perc. (ppmv)	5.7	5.8	5.1	5.1	5.1	5.3	4.7	4.9
99th perc. (ppmv)	7.1	7.3	6.1	6.0	5.9	6.2	5.7	5.7
Maximum (ppmv)	26.3	13.6	15.1	17.0	17.0	10.6	13.1	12.6
>8 ppmv (%)	23.1	32.7	2.0	42.2	9.9	12.6	7.2	70.4
>10 ppmv (%)	36.9	34.6	5.8	23.7	9.8	2.4	17.1	70.7
>12 ppmv (%)	60.7	7.1	14.3	17.9	33.3	0	16.7	50.0
>14 ppmv (%)	77.8	0	11.1	11.1	100	0	0	0
>16 ppmv (%)	80.0	0	0	20	100	0	0	0
>18 ppmv (%)	100	0	0	0	n/a	n/a	n/a	n/a

Figures 1c and 1d illustrate the annual cycle of 100‐hPa H_2_O in the NA region over 2005–2019. Figure 1c shows a time series of monthly histograms (with ∼1,800 profiles each month). The amplitude of the annual cycle is ∼2.2 ppmv around a median of 4.5 ppmv. Outliers are observed between June and August in most years and are particularly obvious in 2007, 2010–2012, and 2016–2019. The fraction of MLS 100‐hPa H_2_O observations exceeding a certain mixing ratio, illustrated as a cumulative histogram summed from the high side, is shown in Figure 1d. June, July, and August exhibit long tails in their distributions with H_2_O > 12 ppmv. However, July and August record the majority of outliers, as 671 of 802 (83.7%), 13 of 17 (76.5%), and 3 of 4 (75.0%) NA profiles with 100‐hPa H_2_O > 8, 12, 16 ppmv, respectively, are observed in these 2 months.

May and June are the only other months in which 100‐hPa H_2_O is observed to exceed 10 ppmv. For both months these high values almost exclusively occur during 2013–2019 (14 of 16 instances) and so were not observed by S2013. This trend is illustrated in Figure 1e, where the H_2_O distributions for three months over 2005–2012 are compared to those over the full study period. The increase in OCE frequency in earlier months after 2012 suggests a strengthening of the NA monsoon.

An increase in frequency of larger NA outliers is apparent throughout the LMS. Figure 1f presents total distributions of H_2_O at 100, 82.5, and 46.4 hPa, again covering 2005–2012 and 2005–2019 over NA. At each level the tails of largest mixing ratios extend further when later years are included.

Similar seasonal evolution and trends (albeit less extreme) for H_2_O observed over AMA and SA are shown in the supporting information (“SI” hereinafter), Figures S1–2.

## 4 Singular H_2_O Profile

Multiple factors instill confidence that the 100‐hPa $H_{2}O=26.3$ ppmv peak value is not a retrieval artifact. First, the MLS data quality metrics indicate successful retrievals for all levels, and qualitative comparisons with other H_2_O > 8 ppmv events in the MLS record reveal that, outside of the elevated values at 83–100 hPa, this profile appears consistent with others. Similarly, the coincident retrievals of N_2_O, O_3_, CO, and HCN are unremarkable. As with many of the >800 profiles in the NA data set that exhibit H_2_O outliers, some of the radiances show signs of possible cloud influence and have been discarded in the Version 4 retrieval; however, the number and distribution of discarded radiances for this profile are typical of other outlier profiles. Finally, an independent 1D retrieval algorithm, based on Livesey and Read (2000), estimates H_2_O > 21 ppmv at 100 hPa. Although this value is lower than the Version 4 estimate (likely because of the algorithm's assumption of horizontal homogeneity along the instrument line‐of‐sight), it is still larger than all the other profiles in the Version 4 record.

**Figure 2:**
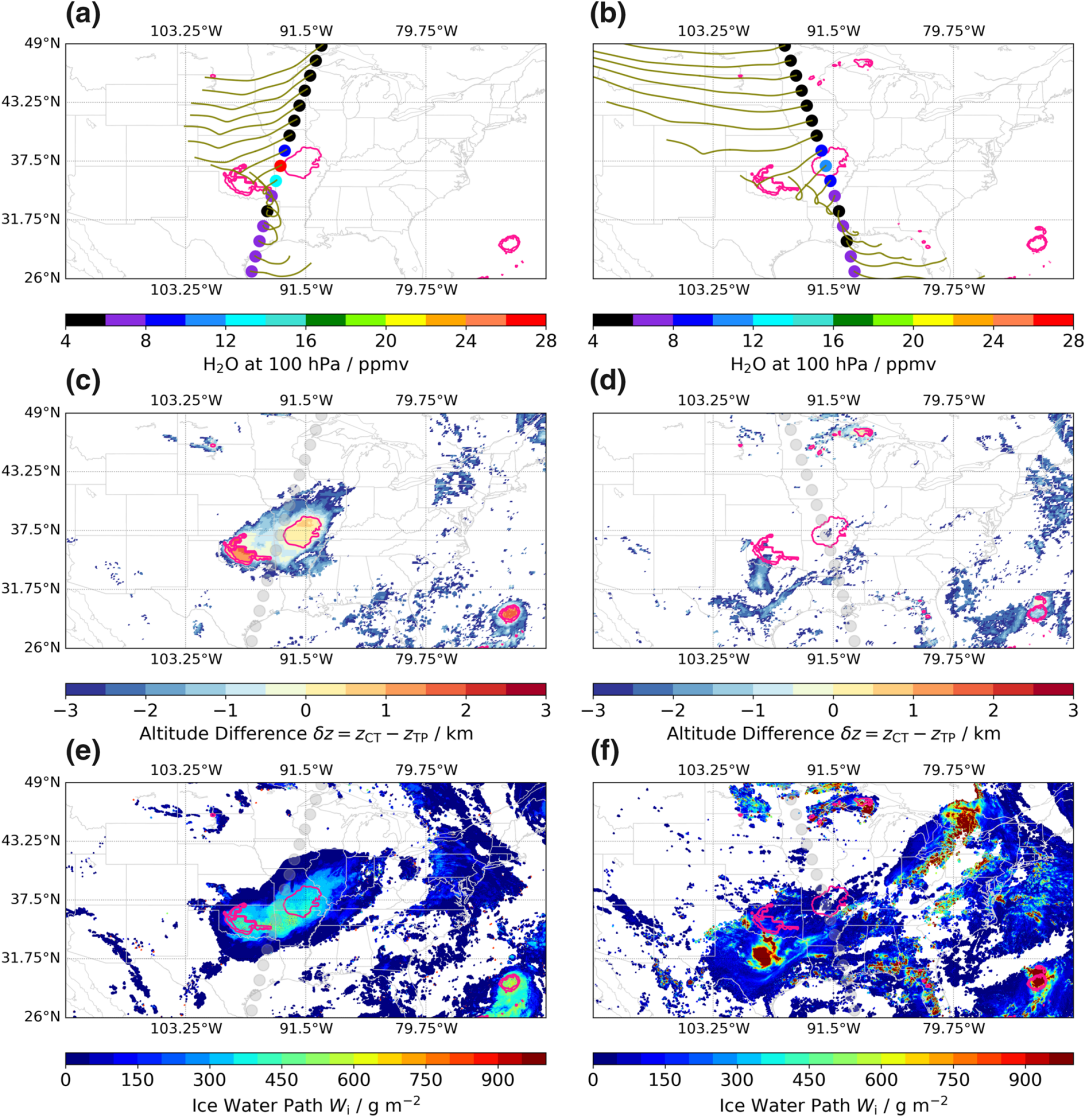
(a) Retrievals of 100‐hPa H_2_O from the descending MLS orbit on 27 August 2019 around 8:20 UTC (dots) and back trajectories for each retrieval (olive lines). (b) As (a), but for the ascending orbit around 19:30 UTC. (c) Values of *δz* for the descending orbit ( δz=0 contours are shown in pink and repeated on all panels). Translucent gray circles illustrate the MLS orbit. (d) As (c), but for the ascending orbit. (e, f) Estimated ice water path (*W*_i_) from GOES‐R observations from the descending and ascending orbit, respectively.

Figure 2 relates the MLS measurements to GOES‐R observations of high‐reaching convection. Figure 2a shows H_2_O from the descending MLS track over NA on 27 August 2019 around 8:20 UTC. The 24‐hr back trajectories indicate the path of the sampled air masses. The GOES‐R‐derived *δz* and *W*_i_ are depicted in Figures 2c and 2e, respectively.

The singular MLS 100‐hPa H_2_O retrieval, depicted in red in Figure 2a, is observed to be coincident with an OCE over Missouri with *δz* ∼ 0.5 km (Figure 2c) and *W*_i_ ∼ 350 g m^−2^ (Figure 2e). The profiles immediately before and after also exhibit high mixing ratios of 9.5 ppmv (dark blue) and 12.9 ppmv (cyan), respectively. In addition, a second OCE (*δz* ∼ 1.5 km and *W*_i_ ∼ 400 g m^−2^) lies upstream of the MLS profile over Oklahoma. These two regions are part of a large‐scale storm that covers multiple states and thousands of square kilometers. The back trajectories for the singular H_2_O event pass through the northeastern corner of Oklahoma, where large positive values of *δz* > 1 km are observed.

GOES‐R observations (see SI, Figure S3) reveal that the two OCEs started to appear between midnight and 1:00 UTC, consistent with Chen and Houze (1997) who show that mesoscale systems typically exhibit maximal cloud top area before dawn. GOES‐R data further show that these OCEs persisted until the time of the subsequent MLS overpass. Thus, not only was the singular profile directly above an OCE, but also water vapor had likely been convectively injected into the LMS for several hours prior to the MLS measurement from both OCEs.

The remnants of the convectively injected water vapor, diluted through mixing in the intervening 11 hours, are observed by MLS during the ascending orbit around 19:30 UTC (Figures 2b, 2d, and 2f). Three profiles with 100‐hPa $H_{2}O\;=\;8.1,\;9.9$, and 11.8 ppmv are observed over Missouri at the position of the prior OCE (depicted by the replicated pink Figures 2a, 2c, and 2e). Moreover, back trajectories indicate that the profiles lie downstream of the prior OCE over Oklahoma. This shows that humidity outliers from earlier events, as well as those advected over large distances, can persist in the LMS in a manner observable by MLS.

## 5 Statistics of Overshooting Convection From Colocated MLS‐MODIS Observations

The size of the OCEs depicted in Figure 2, together with their duration and proximity to MLS profiles, provides a reasonable explanation for the large 100‐hPa H_2_O outliers. However, questions remain: Which properties of this specific event conspired to make it the largest (by far) retrieved H_2_O in the MLS record? Why are similarly large outliers not sampled more often? To better distinguish this event from others, every MLS profile in the NA box during July and August over 2005–2019 is colocated with Aqua‐MODIS cloud variables. This allows for a detailed analysis of (i) the frequency of OCEs over NA and of (ii) correlations between 100‐hPa H_2_O values and characteristics of individual OCEs. The 15‐minutes delay between MODIS and MLS samples is neglected here, as we focus on large convective cloud systems, which can prevail for several hours and are characterized by slow changes in their macrophysical cloud structure (Chen & Houze, 1997).

The data set indicates that 0.4% of the ∼4.7 × 10^8^ sampled MODIS pixels over NA in July and August identify convection above the tropopause (not shown). This frequency is on the same order as the frequency of 100‐hPa H_2_O > 8 ppmv over NA, shown in Figure 1a, with an average of 0.3%.

**Figure 3:**
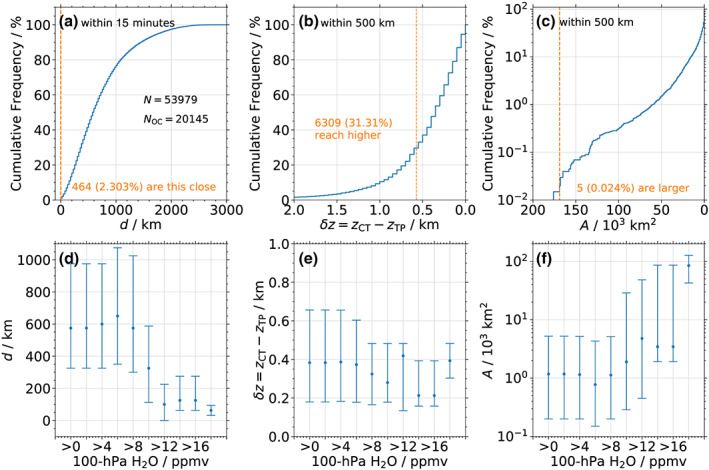
(a) Cumulative histogram of minimum distances (*d*) between individual MLS profiles and MODIS observations that are characterized by an OCE (*δz* > 0) over 2005–2019. The total number of profiles (*N*) and those with *δz* > 0 within 3,000 km (*N*_OC_) are given. The orange dashed line highlights the value corresponding to the singular event on 27 August 2019. (b and c) Similar to (a), but for the median *δz* and area of the overshooting event (*A*), respectively. (d) Median (dots) and interquartile range (vertical lines) of *d* as a function of H_2_O. (e–f) Same as (d), but for *δz* and *A*, respectively.

Figure 3a shows the cumulative histogram, summed from the low side, of the distance (*d*) between each MLS profile and an OCE, if MODIS detects one coinciding with the MLS overpass. Of the N=53,979 MLS profiles in the July/August NA analysis, $N_{\text{OC}}=20,145$ (37.3%) are within *d* = 3,000 km of at least one MODIS pixel with *δz* > 0. Of these observations, 25%, 50%, and 75% are characterized by *d* < 300, 575, and 950 km, respectively. For the singular $H_{2}O=26.3$ ppmv event, d=0 km (indicated by the vertical orange dashed line). Only about 2.3% of MLS profiles in the vicinity of a MODIS pixel with *δz* > 0 are this close.

Figures 3b and 3c similarly illustrate the cumulative histogram of the median *δz* and the area of the overshooting event (*A*) within a 500 km radius of the MLS profile (i.e., twice the MLS along‐track resolution; a 1,000 km radius yields similar results). For *δz*, 25%, 50%, and 75% have median values of 0.7, 0.4, and 0.2 km, respectively, while δz=0.57 km for the singular H_2_O event. Thus, slightly fewer than one third of MLS profiles are in the vicinity of an overshooting convective event that reaches further into the LMS than the case study OCE. At the same time, the singular H_2_O profile is surrounded by one of the largest OCEs in the study period, with $A=168,950\;{\text{km}}^{2}$. Only five other MLS profiles, from only two convective events, are close to OCEs with the same or larger areas: the very next profile after the 100‐hPa $H_{2}O=26.3$ ppmv observation (see Figure 2a), and four consecutive profiles close to a just‐developed OCE in 2009 that are not associated with H_2_O outliers (see SI, Figure S4). Overall, 25% and 50% of OCEs near an MLS profile cover an area larger than 5,000 and 1, 000 km^2^, respectively (note the logarithmic *y axis* in Figure 3c).

Figures 3d–3f show correlations of these OCE metrics with MLS H_2_O. Circles represent the median *d*, *δz*, and *A* within each H_2_O bin, while vertical bars indicate the interquartile range (i.e., the 75th and 25th percentiles). For *d*, no correlation with retrieved mixing ratios is apparent for profiles with H_2_O ≤ 10 ppmv. However, larger outliers coincide with smaller distances, with median values of *d* < 125 km for H_2_O ≥ 12 ppmv and *d* < 62.5 km for H_2_O ≥ 18 ppmv. No correlation between *δz* and H_2_O is observed, as 0.2 km < *δz* < 0.5 km for all mixing ratios. Conversely, a strong relation exists between *A* and H_2_O. While the median *A* is <1,200km^2^ for H_2_O < 10 ppmv, the covered area increases exponentially for larger mixing ratios. This is especially true for the 75th percentile of MODIS‐observed *A*, which reaches values of up to 126,806 km^2^ for H_2_O ≥ 18 ppmv. Note that no correlation between H_2_O outliers and MODIS‐observed *W*_i_ is found (not shown).

## 6 Summary and Conclusions

The S2013 study used global MLS data over 2005–2012 to demonstrate that the largest H_2_O outliers in the LMS are found in the NA region during summer. By using the updated Version 4 MLS products and extending the time series to include seven additional years (2013–2019), this study provides more accurate outlier statistics for the LMS. The updated data set confirms that a majority of 100‐hPa outliers are observed over NA (60.7% for H_2_O > 12 ppmv and 80.0% (four of five) for H_2_O > 16 ppmv), while indicating two trends for the NA region: (i) large H_2_O outliers start to appear earlier in the year during 2013–2019, hinting at a strengthening of the NA monsoon season, and (ii) the probability distributions of H_2_O exhibit an increase in the frequency of larger outliers during 2013–2019. Indeed, the largest NA outlier was sampled in August 2019. This singular profile exhibits $H_{2}O=23.6$ ppmv at 100 hPa, far exceeding the typically observed NA summer enhancements of ∼6.8 ppmv, compared to a median of ∼4.5 ppmv. Both trends warrant future investigation.

By colocating 15 years of MLS H_2_O profiles with cloud products from GOES‐R and MODIS, this study also provides evidence that the largest outliers in the LMS over the NA monsoon coincide with the occurrence of extensive OCEs. A case study for the maximum H_2_O reveals that the record outlier is associated with a convective system covering multiple U.S. states, including two large OCEs that developed from the same large storm several hours before the MLS overpass. The singular MLS retrieval is sampled above one of these areas, and trajectories also show that the air mass at the MLS position lies downstream of the second OCE in the perimeter of the record profile. A second MLS overpass in close proximity to the first orbit about 11 hours later still observed considerably elevated H_2_O of up to 11.8 ppmv, even though the cloud structures had dissipated in the interim.

Our analysis reveals that the close proximity between the OCE and singular MLS profile, as well its covered area, represent rare occurrences during the MLS mission: only ∼2.3% of profiles are similarly close, while ∼0.024% of adjacent OCEs are larger. We find that profiles with larger 100‐hPa H_2_O outliers (e.g., >12 ppmv) are closer to OCEs that cover a larger area. Conversely, no correlations are found between H_2_O outliers and cloud penetration depth into the LMS.

If we consider proximity to an OCE and its area to be independent quantities, only 15(years)·365(days)·3,495(daily profiles)·1.8%(NA coverage)·2.3%·0.024%=1.9 such high H_2_O outliers are expected in the NA data record, which is close to predicting a singular event. If this estimated frequency were extrapolated to the entire globe, 132 similar events would be expected in the MLS record. The lack of such events globally highlights the importance of the NA region with regard to convective moistening of the LMS.

Although MODIS cannot provide information about the temporal evolution of OCEs, GOES‐R data show that the OCE associated with the singular H_2_O event existed for several hours prior to the MLS overpass. It is likely that multiple profiles with low/average H_2_O are similarly close to a just‐developed OCE that has had less time to inject ice into the desiccated stratospheric environment (including the event depicted in SI, Figure S4). Likewise, the H_2_O outliers observed during the second NA orbit 11 hours later were not connected to a concurrent OCE. Instead, MLS probed the remnants of earlier OCEs. This data set alone cannot answer the question of how often MLS outliers are observed downstream of OCEs. More research is necessary to better understand the impact of OCEs on stratospheric H_2_O. Future satellite instrumentation with higher spatiotemporal resolution, as well as extensive radar data sets could provide further insight into these events. If future studies confirm that OCEs noticeably enhance stratospheric H_2_O after large‐scale mixing, these enhancements could be transported into the upper stratosphere via the Brewer‐Dobson circulation and thus impact the climate globally.

## Supporting information



Supporting Information S1

## Data Availability

MLS profiles are available online (at https://mls.jpl.nasa.gov). GOES‐R Series ABI data were obtained from NOAA's CLASS (at https://www.bou.class.noaa.gov/saa/products/search?sub_id=0&datatype_family=GRABIPRD&submit.x=33&submit.y=11). The meteorological fields from MERRA‐2 are available at online (https://disc.gsfc.nasa.gov/datasets?keywords=M2I1NXASM_5.12.4&page=1). Aqua‐MODIS data are from the LAADS‐DAAC (at https://ladsweb.modaps.eosdis.nasa.gov/search/order/1/MODIS:Aqua).
