# Supplementary material for: Extreme Outliers in Lower Stratospheric Water Vapor Over North America Observed by MLS: Relation to Overshooting Convection Diagnosed From Colocated Aqua‐MODIS Data
Source: Geophys Res Lett. 2020 Dec 19;47(24):e2020GL090131. doi: 10.1029/2020GL090131 (PMC7816234; doi:10.1029/2020GL090131)
Supplement: Supplementary file 1 — Supporting Information S1 [file GRL-47-e2020GL090131-s001.pdf]

Supporting Information for "Extreme outliers in lower stratospheric water vapor over North America observed by MLS: Relation to overshooting convection diagnosed from colocated Aqua-MODIS data"

F. Werner[1], M. J. Schwartz[1], N. J. Livesey[1], W. J. Read[1], M. L. Santee[1]

[1]Jet Propulsion Laboratory, California Institute of Technology, 4800 Oak Grove Drive, Pasadena, CA 91109, USA

Contents of this file

1. Text S1 to S4
2. Figures S1 to S4

Introduction The main document describes outliers in lowermost stratospheric (LMS) water vapor (H_2O) mixing ratios measured by the Aura Microwave Limb Sounder (MLS) (Waters et al., 2006; Livesey et al., 2020). A majority of the analysis concentrates on observations over North America (NA), which exhibit increased frequencies of H_2O outliers during the summer months. The Supporting Information first presents time series and cumulative distributions of H_2O over two additional regions, which also show increased summertime outlier frequencies. This information, shown in Figures S1 and S2,

is complementary to the similar plots for NA in Figures 1c–1f of the main manuscript. Subsequently, the Supporting Information presents information pertaining to the evolution of the two largest overshooting convective events (OCEs) in the analyzed dataset (see Section 5 of the main document). The tropopause-relative cloud top altitude (δz) and the area covered by the two OCEs are estimated from cloud top information provided by GOES-R (Greenwald et al., 2016), Aqua's Moderate Resolution Imaging Spectroradiometer (MODIS (Ardanuy et al., 1992; Barnes et al., 1998; Platnick et al., 2003)), and the Modern-Era Retrospective Analysis for Research and Applications, Version 2 (MERRA-2) dataset (Gelaro et al., 2017). The first OCE, shown in Figure S3, persisted for many hours and is associated with H_2O outliers. This fact is mentioned in the main document. The second OCE, shown in Figure S4, had just developed. This fact is also conveyed in the main document and offered as a possible explanation for why no H_2O outliers are observed for this scene.

Text S1 and S2. The annual cycle of 100-hPa H_2O over NA between 2005 and 2019 is shown in Figures 1c-1d of the main document. The annual cycle exhibits an amplitude of 2.3 ppmv around a median of 4.5 ppmv, and July and August are the predominant months when H_2O outliers are observed. Comparing frequency distributions from the time period 2005–2012 to those over 2013–2019 reveals an increase in the occurrences of outliers in May-June (see Figure 1e of the main document) and generally larger tails in the cumulative distributions (i.e., outliers with larger H_2O mixing ratios; see Figure 1f of the main document) in the LMS over NA.

Similar time series and cumulative distributions of H_2O over the Asian summer monsoon area (blue box labeled "AMA" in Figure 1a of the main manuscript; 55–125° E, 20–40° N) and for a smaller area in South America (black box labeled "SA" in Figure 1a of the main manuscript; 40–60° W, 25–40° S) are shown in Figures S1 and S2.

Over AMA, a similar annual cycle of H_2O is seen, with an amplitude of $\sim$ 2.6 ppmv around a median of 4.3 ppmv. The majority of outliers are again observed in July and August, and while the July distributions over both 2005–2012 (i.e., the earlier years studies in Schwartz et al. (2013)) and 2005–2019 (i.e., the full study period) look similar, a larger tail in the June distribution for H_2O>10 ppmv appears when the years 2013–2019 are included. The distributions for May and August look almost identical and have been omitted for legibility. Cumulative distributions of H_2O between 100–68.1 hPa from the periods 2005–2012 and 2005–2019 are very similar. However, a shift towards significantly larger outliers with H_2O>8 ppmv at 46.4 hPa (red lines) occurs when later years are included. Compared to NA, the tropopause is considerably higher over AMA, and the large differences at 46.4 hPa could indicate a similar increase in the frequency of H_2O outliers in the LMS over the Asian summer monsoon region.

Over SA, an annual cycle of $\sim$ 2.1 ppmv around a median of 4.1 ppmv is observed, and the cumulative distributions show a peak in the frequency of H_2O>10 ppmv in January and February (as well as October). Similar to NA and AMA, in the months prior to the maximum occurrence of outliers (October and December for SA; November distributions are similar and have been omitted for legibility) an increase in the frequency of outliers is

observed between 2013 and 2019. Likewise, a shift towards larger H_2O outliers between 82.5 and 68.1 hPa is observed in the total cumulative distributions.

Text S3. Section 4 of the main paper presents a case study of a particularly large overshooting convective event (OCE), including δz at the time of the MLS overpass. Positive values of δz indicate GOES-R cloud top altitudes above the (MERRA-2) tropopause. As noted in the discussion of the case study, two large OCEs over Missouri and Oklahoma developed several hours before the MLS overpass, and, as a result, there was ample time for the ice particles at cloud top to sublimate and yield a maximum H_2O outlier of 26.3 ppmv at 100 hPa. Figure S3 illustrates the evolution of δz over NA on 27 August 2019, between 0:20 and 18:30 UTC. Note that the descending and ascending MLS orbits shown in Figure 2 occurred around 8:25 UTC and 19:25 UTC, respectively (indicated here by the gray transparent circles). A large area of $0\,\text{km} < \delta z < 0.5\,\text{km}$ started developing over Missouri around midnight (see panel for 0:20–0:30 UTC). At 2:20–2:30 UTC, a large connected area with positive δz up to 2 km is visible over Oklahoma and Missouri, growing in size within the next 6 hours. The OCE starts dissipating after 10 UTC, and no $\delta z > 0\,\text{km}$ are visible in the vicinity of the H_2O outliers by 16 UTC (i.e., about 3.5 hours before the ascending MLS orbit).

Text S4. The OCE that covered the largest area in the colocated MLS-MODIS data set occurred on 8 August 2009. No GOES-R cloud products are available for this event. Instead, δz is provided by MODIS and MERRA-2 data. MLS 100-hPa H_2O mixing ratios for the descending MLS orbit around 8:20 UTC are presented in Figure S4a; magenta lines indicate backward trajectories for the 24 hours before the MLS overpass. Values of δz are

shown in Figure S4b. A large OCE, indicated by positive δz up to 2.5 km, is visible over Wisconsin and directly underneath the MLS overpass around 8:20 UTC. However, the profiles associated with that OCE exhibit no outliers at 100 hPa, and all $H_2O<6$ ppmv. Two of the back trajectories pass through the smaller areas of positive δz upstream of the MLS overpass, but these smaller OCEs had not developed yet at the respective times.

MODIS only provides a snapshot of the cloud situation $\sim$ 15 minutes before the MLS overpass. To understand the evolution of the large OCE over Wisconsin, a time series of hourly band 5 brightness temperatures (T_{B5}) sampled by GOES-11 are shown in Figure S4c. Note that this dataset can only provide a quantitative perspective on cloud top altitude and the conclusions on overshooting conditions are somewhat speculative. The OCE at 8 UTC (i.e., shortly before the MLS overpass) shows cold temperatures of $\sim$ 200 K. This area of low T_{B5} is first visible as a thin strip east of the MLS overpass around 6 UTC and covered a larger area by 7 UTC. Compared to the OCE shown in Figure S3, which developed about 8 hours before the MLS overpass, this event developed much closer to the time of the MLS measurements. This shorter amount of time was apparently not sufficient for the ice particles at cloud top to evaporate and substantially increase the humidity in the LMS.

References

Ardanuy, P. A., Han, D., & Salomonson, V. V. (1992). The Moderate Resolution Imaging Spectrometer (MODIS). *IEEE Trans. Geosci. Remote Sensing*, *30*, 2–27.

Barnes, W. L., Pagano, T. S., & Salomonson, V. V. (1998). Prelaunch characteristics of the 'Moderate Resolution Imaging Spectroradiometer' (MODIS) on EOS–AM1.

IEEE Trans. Geosci. Remote Sensing, *36*, 1088–1100.

Gelaro, R., McCarty, W., Suárez, M. J., Todling, R., Molod, A., Takacs, L., ... Zhao, B. (2017). The Modern-Era Retrospective Analysis for Research and Applications, Version 2 (MERRA-2). *Journal of Climate*, *30*(14), 5419-5454. doi: 10.1175/JCLI-D-16-0758.1

Greenwald, T. J., Pierce, R. B., Schaack, T., Otkin, J., Rogal, M., Bah, K., ... Huang, H.-L. (2016). Real-Time Simulation of the GOES-R ABI for User Readiness and Product Evaluation. *Bulletin of the American Meteorological Society*, *97*(2), 245-261. doi: 10.1175/BAMS-D-14-00007.1

Livesey, N. J., Read, W. G., Wagner, P. A., Froidevaux, L., Lambert, A., Manney, G. L., ... Lay, R. R. (2020, April). *Version 4.2x Level 2 and 3 data quality and description document.* (Tech. Rep. No. JPL D-33509 Rev. E). Pasadena, California, 91109-8099: Jet Propulsion Laboratory, California Institute of Technology.

Platnick, S., King, M., Ackerman, S., Menzel, W., Baum, B., Riedi, J., & Frey, R. (2003). The MODIS cloud products: Algorithms and examples from TERRA. *IEEE Trans. Geosci. Remote Sens.*, *41*, 459-473.

Schwartz, M. J., Read, W. G., Santee, M. L., Livesey, N. J., Froidevaux, L., Lambert, A., & Manney, G. L. (2013). Convectively injected water vapor in the North American summer lowermost stratosphere. *Geophysical Research Letters*, *40*(10), 2316-2321. doi: 10.1002/grl.50421

Waters, J. W., Froidevaux, L., Harwood, R. S., Jarnot, R. F., Pickett, H. M., Read, W. G., ... Walch, M. J. (2006). The Earth Observing System Microwave Limb

Sounder (EOS MLS) on the Aura satellite. *IEEE Transactions on Geoscience and Remote Sensing*, *44*(5), 1075-1092. doi: 10.1109/TGRS.2006.873771

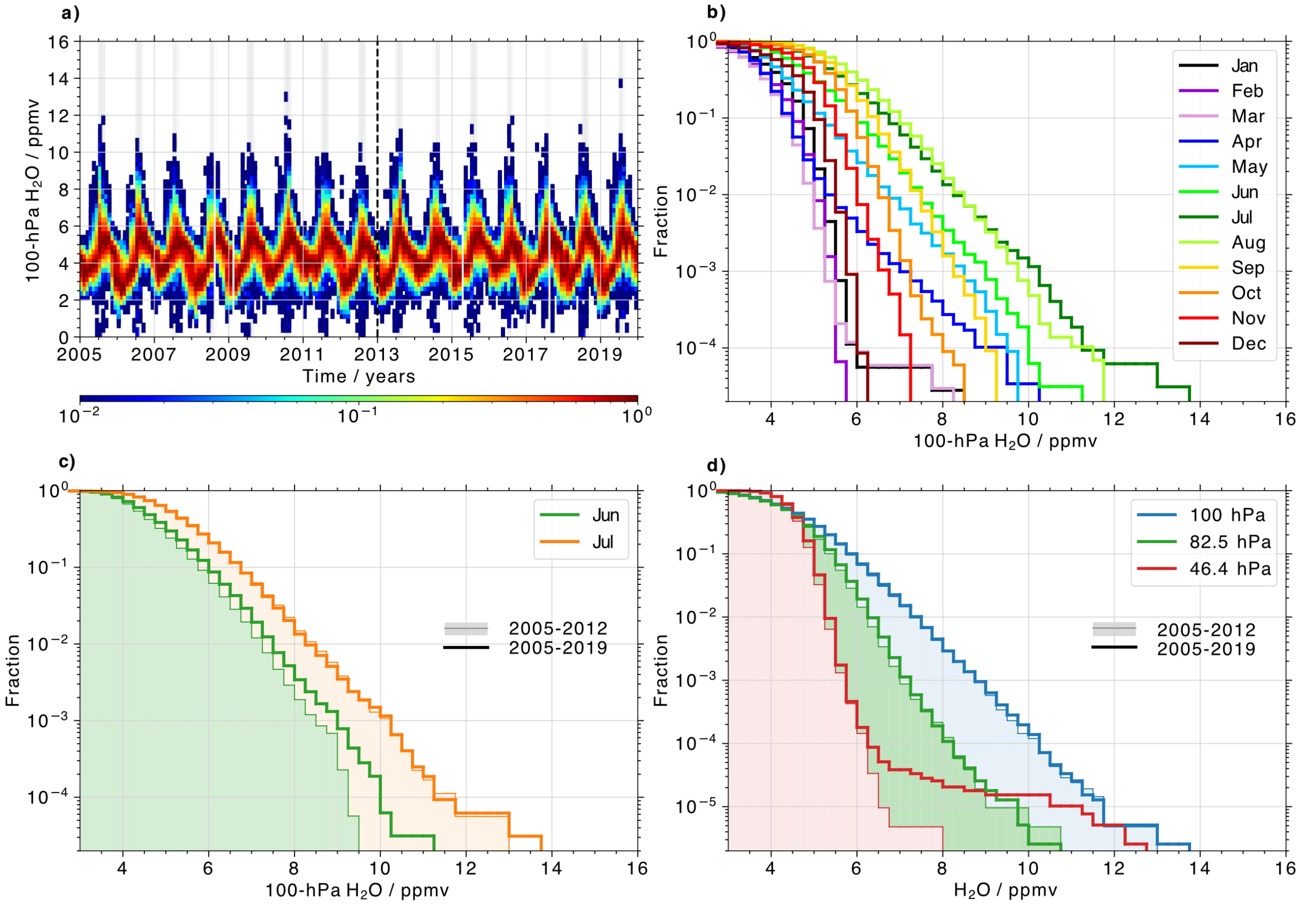

Figure S1. (a) Time series of monthly histograms (each normalized by the maximum) of 100-hPa H_2O in the AMA box over 2005–2019. Gray shading indicates July and August. (b) Monthly probabilities that 100-hPa H_2O observations exceed a given mixing ratio. (c) Same as (b), but comparing June-July probabilities between the earlier years (2005–2012; translucent dotted lines) and the full study period (2005-2019; solid lines). (d) Probabilities that lower stratospheric H_2O between 100–46.4 hPa exceed a given mixing ratio over 2005–2012 (translucent shading) and 2005–2019 (solid lines).

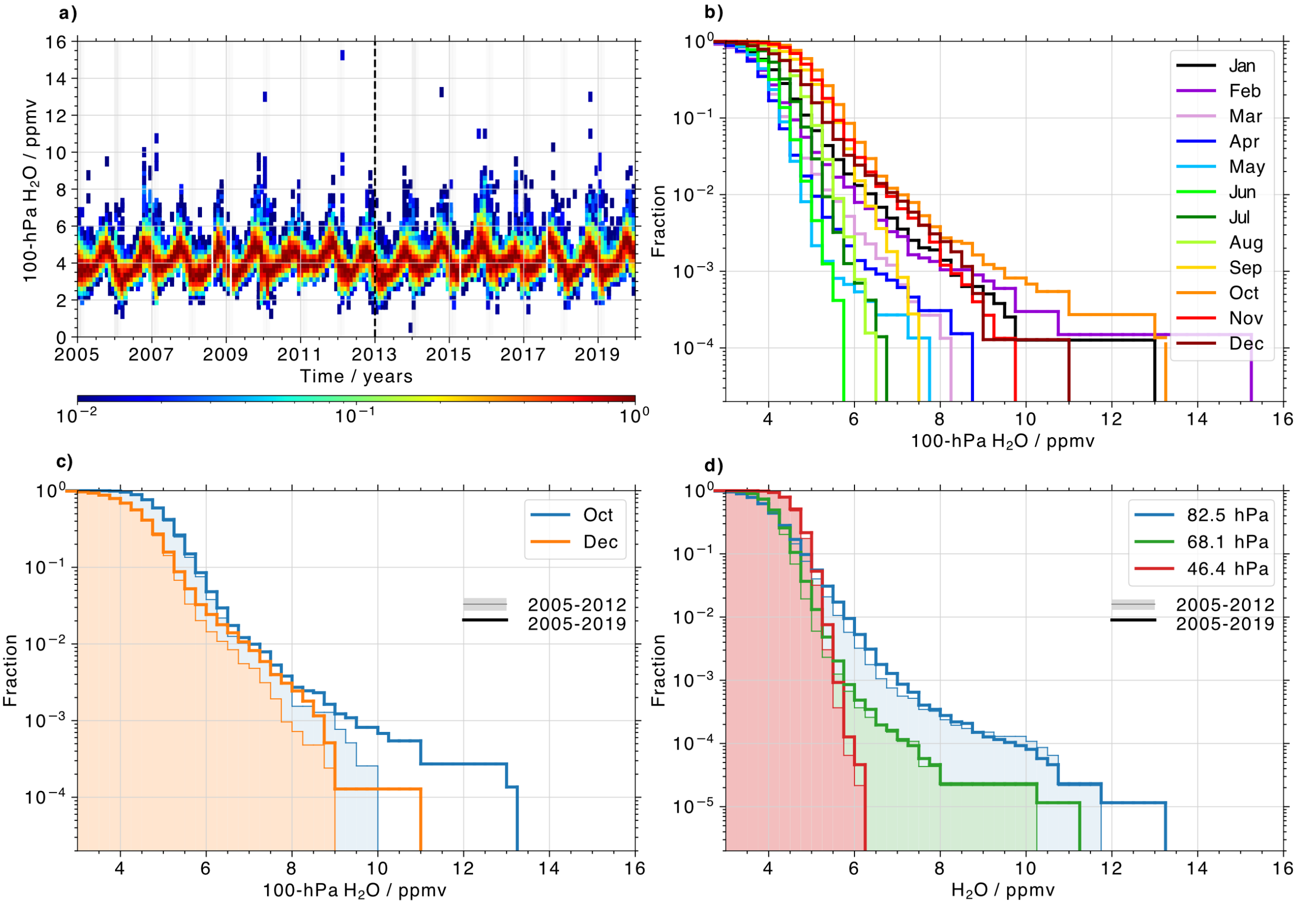

Figure S2. (a) Time series of monthly histograms (each normalized by the maximum) of 100-hPa H_2O in the SA box over 2005–2019. Gray shading indicates January and February. (b) Monthly probabilities that 100-hPa H_2O observations exceed a given mixing ratio. (c) Same as (b), but comparing October-December probabilities between the earlier years (2005–2012; translucent dotted lines) and the full study period (2005-2019; solid lines). (d) Probabilities that lower stratospheric H_2O between 100–46.4 hPa exceed a given mixing ratio over 2005–2012 (translucent dotted lines) and 2005–2019 (solid lines).

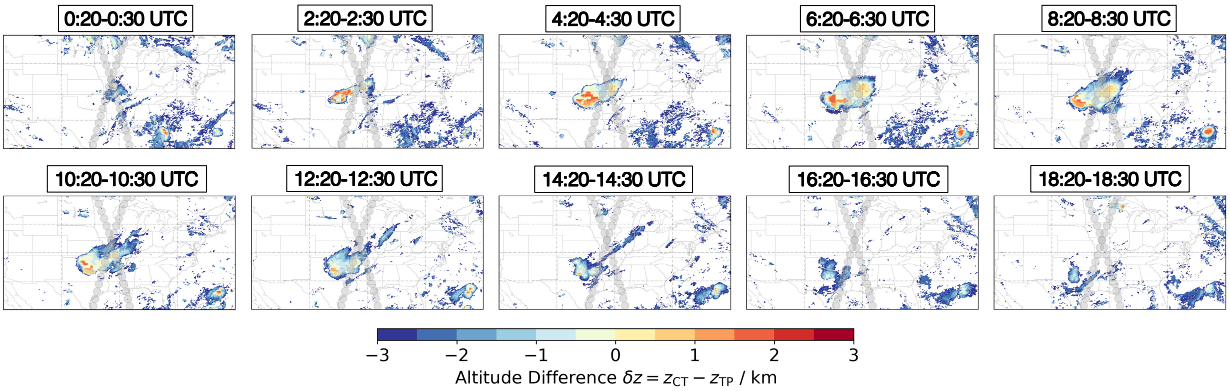

Figure S3. Time series of δz for the overshooting convective event on 27 August 2019 (shown in Figure 2 in the main document).

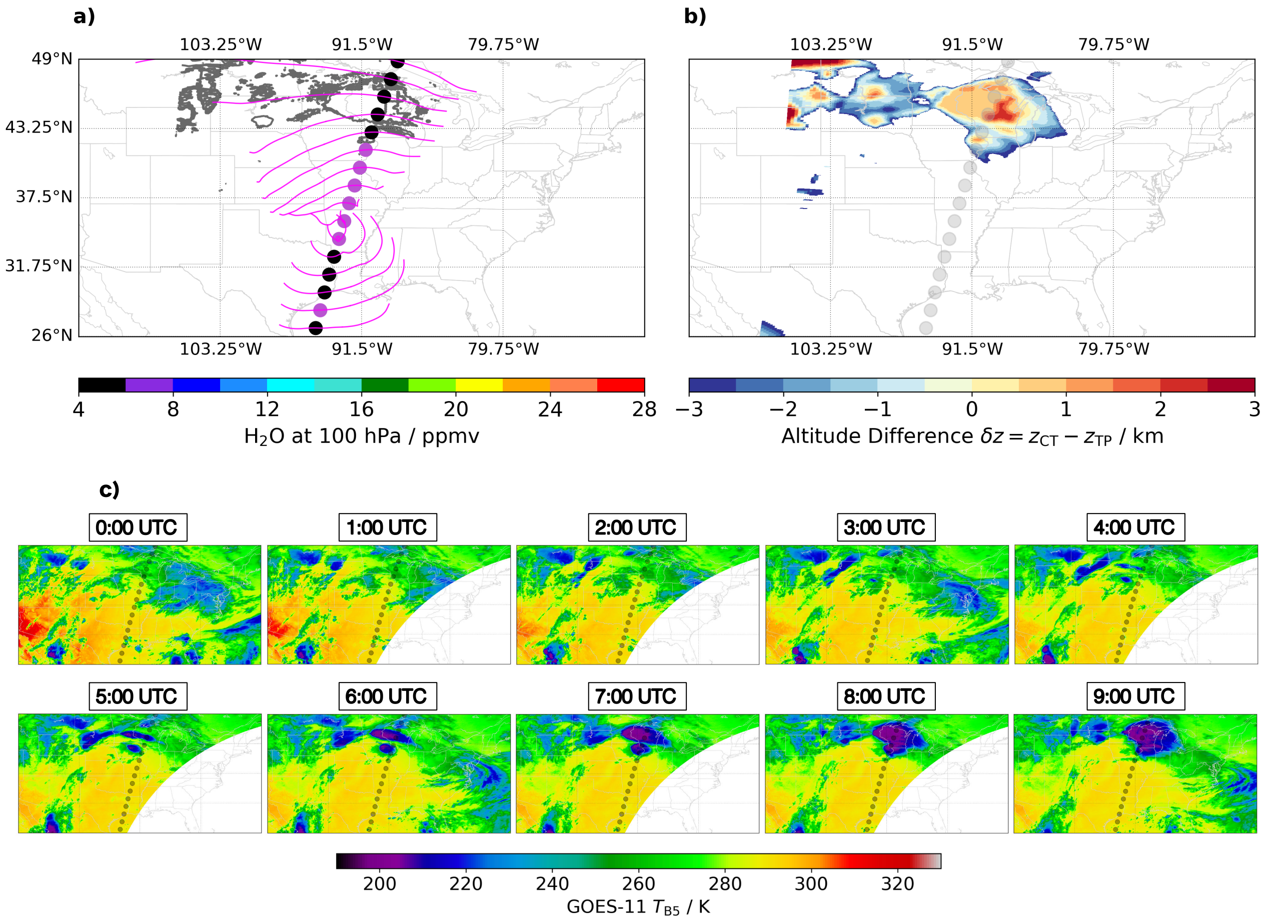

Figure S4. (a) Retrievals of 100-hPa H_2O from the descending MLS orbit on 8 August 2009 around 8:20 UTC (dots) and 24-hour back trajectories for each profile (magenta lines). (b) Values of MODIS-derived δz on 8 August 2009 around 8:20 UTC. (c) Time series of hourly band 5 brightness temperatures (T_{B5}) sampled by GOES-11 on 8 August 2009, covering the time before the MLS overpass around 8:20 UTC.
